# Effect of Bradykinin Postconditioning on Ischemic and Toxic Brain Damage

**DOI:** 10.1007/s11064-015-1675-1

**Published:** 2015-07-28

**Authors:** Mária Lalkovičová, Petra Bonová, Jozef Burda, Viera Danielisová

**Affiliations:** Department of Neurochemistry, Institute of Neurobiology, Slovak Academy of Sciences, Šoltésovej 4-6, 04001 Kosice, Slovak Republic

**Keywords:** Ischemia, Post-conditioning, Glutamate, Bradykinin, Intoxication

## Abstract

Brain damage caused by ischemia or toxic agents leads in selectively vulnerable regions to apoptosis-like delayed neuronal death and can result in irreversible damage. Selectively vulnerable neurons of the CA1 area of hippocampus are particularly sensitive to ischemic damage. We investigated the effects of bradykinin (BR) postconditioning on cerebral ischemic and toxic injury. Transient forebrain ischemia was induced by four-vessel occlusion for 10 min and toxic injury was induced by trimethyltin (TMT, 8 µg/kg i.p.). BR as a postconditioner at a dose of 150 µg/kg was applied intraperitoneally 48 h after ischemia or TMT intoxication. Experimental animals were divided into groups according to the length of survival (short—3 and 7 days, and long—28 days survival) and according to the applied ischemic or toxic injury. Glutamate concentration was lowered in both CA1 and dentate gyrus areas of hippocampus after the application of BR postconditioning in both ischemic and toxic brain damage. The number of degenerated neurons in the hippocampal CA1 region was significantly lower in BR-treated ischemic and toxic groups compared to vehicle group. The behavioral test used in our experiments confirms also the memory improvement in conditioned animals. The rats’ ability to form spatial maps and learn was preserved, which is visible from our Barnes maze results. By using the methods of delayed postconditioning is possible to stimulate the endogenous protective mechanisms of the organism and induce the neuroprotective effect. In this study we demonstrated that BR postconditioning, if applied before the onset of irreversible neurodegenerative changes, induced neuroprotection against ischemic or toxic injury.

## Introduction

Glutamate is the principal excitatory neurotransmitter in the nervous system of vertebrates. It is an essential agent in the processes of long-term neuronal potentiation, learning and retaining memory. It is an excitatory amino acid but also a potent neurotoxin, and glutamate excitotoxicity has been identified in many neurological diseases, including Alzheimers disease and cerebral ischemia [1]. It is a critical transmitter for signaling neurons to degenerate following stroke. Glutamic acid is the most abundant excitatory neurotransmitter in the mammalian CNS, present at perhaps one-third of all rapid excitatory synapses in the CNS [2]. Apart from glutamic acid itself, the excitatory amino-acid class includes aspartic acid, as well as exogenous compounds of natural (quisqualic, kainic, and domoic acids) or synthetic origin [N-methyl-D-aspartic acid (NMDA)]. Glutamic-acid group of excitants have actions which are non-specific with respect to neuronal type and exciting many functionally different types of neuron, such as cholinoceptive and non-cholinoceptive cells in the spinal cord [3], brainstem [4] and cerebral cortex [5]. In normal synaptic functioning, the excitatory action of glutamic acid is rapidly terminated due to its efficient removal from the synapse by glutamic acid uptake systems in glia and nerve terminals [6]. This function is performed by specific transporter proteins that allow co-transport of glutamic acid with sodium ions and concomitant counter-transport of potassium ions, using the transmembrane electrochemical gradient for sodium and potassium as the driving force. This process is highly efficient and enables glutamic acid to be concentrated in the intracellular compartment up to 10,000-fold with respect to the extracellular milieu. Extracellular glutamic acid concentrations can be thus kept at levels of around 1 mM. Glutamate-induced neurotransmission is mediated through ionotropic glutamate receptor subtypes, such as N-methyl-D-aspartic acid (NMDA), *α*-amino-3-hydroxy-5-methylisoxazole-4-propionic acid and kainate receptors [7]. During the normal physiological process of rapid excitatory synaptic transmission, glutamic acid is released from glutamatergic nerve terminals in response to depolarization, crosses the synaptic cleft, and acts on postsynaptic receptors. These receptors are membrane ion channels, and their activation leads to the entry of cations into the postsynaptic neurone and subsequent depolarisation. When depolarization of the neuronal membrane reaches a certain threshold, a train of action potentials is generated.

Excitotoxicity refers to a process of neuronal death caused by excessive or prolonged activation of glutamic acid receptors. For over a decade, researchers have been trying to treat neurodegenerative diseases with drugs that block excitotoxicity. That there is indeed a massive rise in the extracellular concentrations of glutamic acid following cerebral ischaemia has been demonstrated by microdialysis techniques in rats subjected to a transient global cerebral ischemia [8].

The hippocampus has a predeliction for ischemic injury of the selective vulnerability type, especially its CA1 sector. In selectively vulnerable neurons, a delayed cell death that appears 2–3 days after a lethal stress is observed [9, 10]. This provides a therapeutic window for application of a second, sub-lethal stress, that can induce tolerance of cells after the lethal stress. This delayed post-conditioning also lowers glutamate levels after ischemic injury and improves protein synthesis in the CA1 area and the dentate gyrus [11, 12].

The application of pharmacological stressors such as 3-nitropropionic acid, norepinephrine or bradykinin, can induce neuroprotection [13–15]. Inhibition of glutamic acid release by riluzole has also been described neuro-protective in rat hippocampal slices [16]. Bradykinin (BK) is an endogenous peptide and has been studied for its ability to act as a mediator of brain damage in acute insults. It is known to influence the inflammatory process affecting various tissue reactions, such as peripheral vasodilatation, and to increase vascular permeability. BK activation can alter the permeability of cerebral vessels during cerebral ischemia and may therefore influence the progression of ischemic edema [17]. After brain trauma or stroke, BK production is upregulated and the increased BK levels lead to an increase in blood–brain barrier permeability and an accumulation of leukocytes [18, 19]. BK is thus thought to be involved in secondary brain damage. By blocking BK receptors with specific antagonists, post-ischemic brain swelling after focal cerebral ischemia is reduced and functional neuronal recovery is improved [20]. Trimethyltin (TMT) is an organic tin compound that has been reported to cause effects in humans that include memory loss, anorexia, disorientation, rage reactions, photo sensitivity, depression and epileptic seizures [21–23]. TMT causes a behavioral syndrome in rats consisting of hyper-reactivity, tail mutilation and seizures [24]. The effects on aggression and memory are primarily due to loss of septal structures and extensive destruction of neurons in the amygdala and hippocampus. In mice and rats TMT was shown to induce lesions in the hippocampus and impair spatial memory [25]. It could cause hippocampal damage by a direct neurotoxic effect and/or as a result of the seizures it induces. TMT-induced seizures were associated with a variable pattern of granule and pyramidal cell necrosis and acute dendritic swelling in the two associational/commissural hippocampal pathways, one from CA3 to CA1–CA3 and the other from the hilus to the proximal dendrites of dentate granule cells [26]. Intoxication with TMT leads to profound behavioral and cognitive deficits in both humans [27] and experimental animals [28–30]. The neurotoxic presentation most frequently reported in human cases is a limbic-cerebellar syndrome, the manifestations of which include memory defects, confusion, seizures, tinnitus, insomnia, and depression [31]. In mice and rats TMT intoxication induced lesions in the hippocampus and impair spatial memory [25]. It was observed that ROS were elevated immediately in hippocampal neuronal cells (HT-22 cell) after TMT treatment [32]. TMT exposure induced cell apoptosis in fish brain. In addition, TMT exposure elevated the contents of ROS and NO, and the total activities of NOS and iNOS. NO, reported as an important inducer of apoptosis, also plays a considerable role in the mechanisms of TMT toxicity. Studies of the complex molecular and biochemical mechanisms involved in apoptosis have revealed that cell apoptosis involves the overproduction of NO [33, 34]. ROS increase and NO over generation via induction of iNOS are likely to be involved in the apoptosis of brain cells induced by TMT. TMT enhances formation of reactive oxygen species, alters serotonergic and noradrenergic systems, activates protein kinase C in culture, induces the apoptotic cascade, and activates astrocytes and microglia, resulting in the production of proinflammatory cytokines [35]. Several studies support the hypothesis that TMT induces cell death, particularly in the limbic system. While the mechanisms by which TMT induces neurodegeneration are still not understood, many hypotheses suggest that unwanted neuronal apoptosis could be largely due to calcium overload [36].

Since BK has already been successfully used in postconditioning for treatment of ischemic damage, our interest lays in the study of the effect of its application in ischemia and toxic damage and of the changes in glutamate concentration in brain tissue. We studied the effect of BK application as a second pathophysiological stressor after the ischemic or toxic brain damage by TMT and its possible neuro-protective effect on the selectively-vulnerable neurons of the CA1 area of the hippocampus.

## Materials and Methods

The experiments were approved by the Institutional Ethical Committee in accordance with current national legislation. Adult male albino Wistar rats of approximate weight 300–350 g were used in the experiments. They were maintained on a 12 h light/dark cycle and given food and water ad libitum. The animals were divided into groups according to the stressor used, in each group were 6 experimental animals. The first group was the control (untreated). For the second group, a 10-min global ischemia was applied followed by 3, 7 and 28 day period of reperfusion. In the third group the stressor trimethyltin (8 µg/kg i.p., Sigma-Aldrich, St. Louis, MO, USA) was applied followed by the same reperfusion pattern. As a postconditioner bradykinin (150 µg/kg i.p., Sigma-Aldrich, St. Louis, MO, USA) was applied 2 days after the insult in both ischemic groups. An additional group of animals was intoxicated by injection of kainic acid (KA, 8 mg/kg i.p., Sigma-Aldrich, St. Louis, MO, USA). Kainic acid was dissolved in saline to 4.0 mg KA/ml just prior to use.

### Global Ischemia

In the group where 10 min global ischemia was performed, the animals were subjected to a four-vessel occlusion of transient forebrain global ischemia according to the Pulsinelli model [37]. The rats were anesthetized with chloral hydrate (300 mg/kg i.p., Sigma-Aldrich, St. Louis, MO, USA) and both vertebral arteries were electrocauterized with a monopolar coagulator through the alar foramens of the first cervical vertebra. Both common carotid arteries were exposed through the ventral midline cervical incision and ligatures were placed loosely around each artery without interrupting the carotid blood flow. The animals were allowed to recover from anesthesia. On the next day, under light fluorothane anesthesia (0.5 %), both common carotid arteries were re-exposed and occluded with aneurysmal clips to induce ischemia. After 10 min of bilateral carotid occlusion, blood flow was restored by releasing the clips. During ischemia, rats were placed on a heating thermo pad to maintain a constant body temperature of 37 ± 0.5 °C. The body temperature was controlled by a thermistor placed deeply in the rat ear. Neurological investigations were performed to verify ischemic severity.

### Barnes Maze

For the behavioral testing we used the methods of the Barnes maze by Barnes [38]. The Barnes maze consists of an experimental platform with circular holes around its circumference. Below the surface is an “escape box” which can be reached by the rodent through the corresponding hole on the table top. The model is based on rodents’ aversion to open spaces, which motivates the test subject to seek shelter in the escape box. At first, rats were trained on day one. During the training phase rats were placed on the circular platform for an adaptation period, after which the training period followed. On the next day they were tested and afterwards sacrificed. We measured the time necessary to find the escape box after the training in every experimental group and compared the results.

### Concentration of Glutamic Acid in Nervous Tissue

After decapitation under halothane anaesthesia brains were quickly removed and maintained at 0 °C. During tissue separation the hippocampus was removed and divided into the CA1 region, dentate gyrus and the rest of the hippocampus under a dissecting microscope. Collected tissue was weighted and homogenized in a glass-Teflon homogenizer (5 strokes, 800 rpm, 4 °C) in homogenization buffer (20 mM Tris–HCl pH 7.5 containing 1 mM DTT, 50 mM magnesium acetate, 140 mM KCl, 1 mM EDTA, 2 mM EGTA with addition of Protease Inhibitor Cocktail Tablets, Roche, Germany), and centrifuged at 12,000*g* (15 min, 4 °C). These post-mitochondrial supernatants were aliquoted and frozen at −80 °C until analysis. Total protein concentrations in samples were determined using the Bradford method [39]. For establishing the standard curve, we used BSA. Afterwards, concentrations of glutamic acid in the post-mitochondrial supernatants were measured by a modified enzymatic-fluorimetric method [40] based on an assay described by Graham and Aprison [41]. The concentrations were determined by fluorimetric detection of NADH resulting from the reaction of glutamate and NAD+ catalysed by glutamate dehydrogenase. The glutamate concentration is directly proportional to the concentration of NADH in a reaction. Briefly, 10 µl of supernatant was pipetted into a black 96-well plate and 190 µl of reaction buffer (0.25 M hydrazine hydrate/0.3 M glycine buffer, pH 8.6) containing 200 nM NAD+ and 15 U of glutamate dehydrogenase was added. After 30 min of incubation at RT, the fluorescence intensity of the final product (NADH) was read on a Synergy™ 2 Multi-Mode Microplate Reader (BioTek) at 460 nm with an excitation wavelength 360 nm. Final concentration of glutamate in tissue samples was normalized in accordance with the total protein concentration (nmol/mg of protein) and expressed as a percentage difference from the mean value of glutamate concentration in the sham control group (baseline).

### Flouro Jade B Staining

Transcardiac perfusion was performed under chloral hydrate anesthesia (300 mg/kg, i.p., Sigma-Aldrich, St. Louis, MO, USA). At first, the left ventricle was perfused and the blood vessels washed out with 200 ml of 0.9 % NaCl. Fixation was then performed with 4 % (g/l) paraformaldehyde solution in phosphate buffer saline. Brains were removed, and postfixed overnight in the same solution. After the fixation were brains put into the sucrose solution (30 %) for cryoprotection and afterwards the tissue was sectioned on cryostat. The 33 µm coronal sections of the brain were prepared at the level of bregma −3.3 ± 0.2 mm for hippocampus and randomly selected for Fluoro Jade B staining. This was used to label all degenerating neurons present in the CA1 region of hippocampus. The sections were mounted on 2 % gelatin-coated slides and then dried on a slide warmer at 50 °C for 30 min. The slides were then immersed in a solution containing 1 % sodium hydroxide in 80 % alcohol for 5 min. Slices were then immersed for 2 min in 70 % alcohol and 2 min in distilled water. The slides were then transferred to a solution of 0.06 % potassium permanganate for 10 min, and subsequently rinsed in distilled water for 2 min. After 20 min in the staining solution, containing 0.0004 % Fluoro Jade B dye (Histo-Chem Inc., USA), the slides were rinsed three times for 1 min in distilled water. We briefly drained the slides vertically on a paper towel. The slides were then placed on a slide warmer set at approximately 50 °C until they were fully dry. The dry slides were cleared by immersion in xylene for at least a minute before applying a coverslip with DPX Mountant for histology (Fluka Chemie AG, Switzerland). The slides were examined using an Olympus BX 51 fluorescent microscope with digital camera DP 50 (Olympus Optical CO. LTD, Japan). Neuronal cell count was performed by a blind observer using Image tool software (UTHSCSA, San Antonio, USA). Numbers of Fluoro Jade B-positive CA1 neurons were counted in the middle of the linear part of the CA1and expressed as the average of 10 measurements of positive neurons per 1 mm of the hippocampal CA1 region.

### Immunostaining for NeuN Antibody

Animals were sacrificed after 7 days of reperfusion that followed 10 min ischemia or ischemia + BK postconditioning. The nervous tissue sections were prepared by the same procedure on cryostat and randomly selected for immunocytochemistry, immunostaining for NeuN (Neuronal Nuclei, Neuron-Specific Nuclear Protein), a neuronal biomarker, in the CA1 region of hippocampus. The sections were incubated overnight at 4 °C with NeuN antibody (Chemicon International, Temecula, USA, 1:400) in 0.1 mol/l PBS (pH 7.4) with 0.2 % Triton. In the morning they were washed with 0.1 mol/l PBS (pH 7.4) with 0.2 % Triton, and the secondary anti-mouse IgG antibody was applied for 90 min at room temperature. The sections were washed again, and ABC Elite (Vector Laboratories, Burlingame, USA) was applied for 90 min. The slides were then rinsed with PBS followed by Tris Buffer (pH 7.6), and reacted with DAB (0.1 mol/l Tris, 0.04 % DAB, 0.033 % H2O2); the reaction was stopped with phosphate buffer. The slides were dehydrated, cleared and a coverslip applied for analysis.

### Statistical Analysis

Data are expressed as mean ± SD. Statistical analysis was performed with one-way ANOVA followed by a post hoc Duncan’s test. The value of *p* less than 0.05 was considered to be statistically significant.

## Results

We examined the changes in concentration of glutamate and the effect of delayed postconditioning with bradykinin in nervous tissue. We divided animals into 3 main experimental groups: the control group, the group with 10-min global ischemic insult and the trimethyltin insulted group. Animals in the first subcategory survived for 3, 7 and 28 days without any postconditioning and in the second one bradykinin was applied as a postconditioner 48 h after ischemic or chemical insult.

### Ischemia

**Fig. 1:**
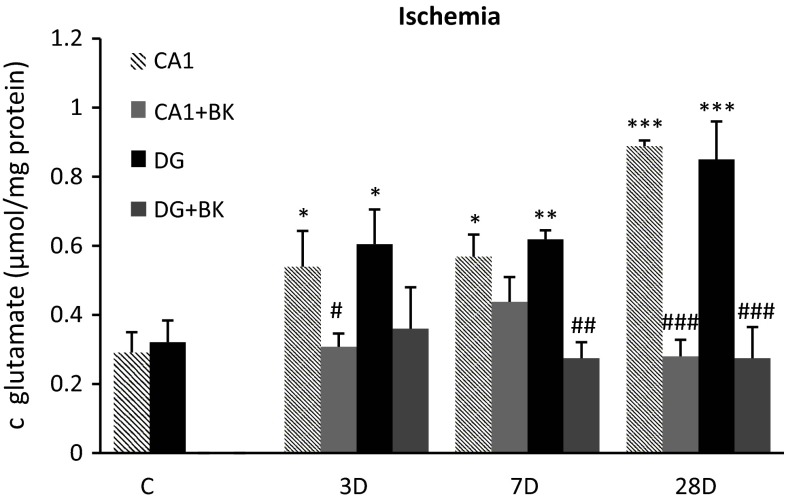
Concentrations of glutamate in CA1 and DG regions of hippocampus after 10-min global ischemia and reperfusion, and after ischemia and application of delayed postconditioning with bradykinin (150 µg/kg i.p.). Mean ± SD, *p* < 0.05 (*, #), *p* < 0.01 (**, ##), *p* < 0.001 (***, ###); *value compared to control, #value compared to ischemia without bradykinin postconditioning, C—control group, BK—bradykinin postconditioning 48 h after ischemia, 3D—experimental group with 10 min ischemia followed 3 day reperfusion, 7D—experimental group with 10 min ischemia followed 7 day reperfusion, 28D—experimental group with 10 min ischemia followed 28 day reperfusion. Concentration of glutamate is expressed in μmol per mg of protein content

The level of glutamate concentration in the control group in CA1 area was 0.291 ± 0.059 µmol/mg of protein. After 10-min ischemia and 3 days of reperfusion, bradykinin postconditioning-reduced glutamate levels from 0.53 ± 0.104 µmol/mg of protein (*p* < 0.05) to 0.308 ± 0.038 µmol/mg of protein (*p* < 0.05). For 28 days of reperfusion, levels went from of 0.888 ± 0.017 µmol/mg of protein (*p* < 0.001) to 0.287 ± 0.048 µmol/mg of protein (*p* < 0.001). In the DG we also observed significant changes in glutamate concentration. With 10-min of ischemia and 7 days of reperfusion, a glutamate level of 0.619 ± 0.026 µmol/mg of protein (*p* < 0.01) was observed, which was higher than both the control value (0.321 ± 0.063 µmol/mg of protein) and that observed with bradykinin postconditioning 0.259 ± 0.046 µmol/mg of protein (*p* < 0.01). With 28 days of reperfusion glutamate was 0.857 ± 0.11 µmol/mg of protein (*p* < 0.001) but was reduced by postconditioning to 0.274 ± 0.09 µmol/mg of protein (*p* < 0.001) (Fig. 1).

### TMT

**Fig. 2:**
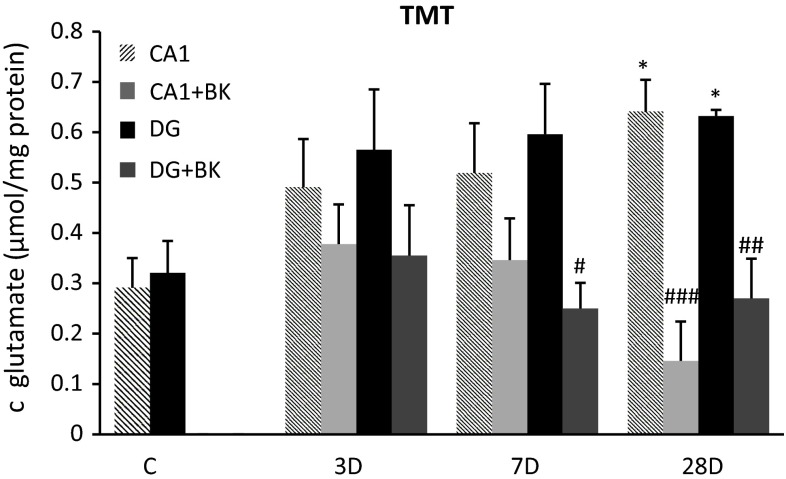
Concentrations of glutamate in CA1 and DG regions of hippocampus after application of TMT (8 µg/kg i.p.) and reperfusion, and following bradykinin postconditioning (150 µg/kg i.p.). Mean ± SD, *p* < 0.05 (*, #), *p* < 0.01 (**, ##), *p* < 0.001 (***, ###); *value compared to control, #value compared to ischemia without bradykinin postconditioning, C—control group, BK—bradykinin postconditioning 48 h after ischemia, 3D—experimental group with TMT injection followed 3 day reperfusion, 7D—experimental group with TMT injection followed 7 day reperfusion, 28D—experimental group with TMT injection followed 28 day reperfusion. Concentration of glutamate is expressed in μmol per mg of protein content

With 28 days of reperfusion after TMT injection, glutamate increased in the CA1 area to 0.641 ± 0.061 µmol/mg of protein (*p* < 0.05) but decreased to 0.146 ± 0.078 µmol/mg of protein (*p* < 0.001) with postconditioning. In the DG after 7 days of reperfusion, glutamate was 0.596 ± 0.1 µmol/mg of protein and was reduced to 0.259 ± 0.051 µmol/mg of protein (*p* < 0.05) by postconditioning. With 28 days of reperfusion glutamate was 0.632 ± 0.012 µmol/mg of protein (*p* < 0.05) and after postconditioning was 0.280 ± 0.079 µmol/mg of protein (*p* < 0.01) (Fig. 2).

### Behavioral

**Fig. 3:**
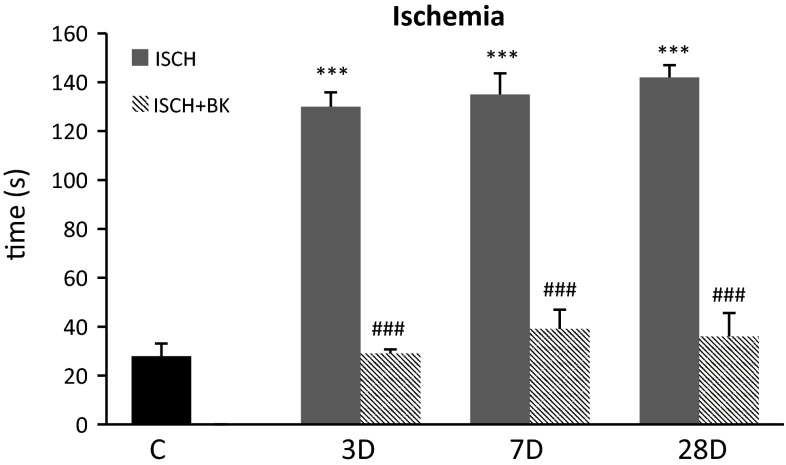
Behavioral results using Barnes Maze. The animals after 10 min of global ischemia were trained 1 day prior to the decapitation and tested on the day of decapitation. The latency to enter the box under the surface of the platform was measured in all experimental groups. Mean ± SD, *p* < 0.05 (*, #), *p* < 0.01 (**, ##), *p* < 0.001 (***, ###); *value compared to control, #value compared to ischemia without bradykinin postconditioning (150 µg/kg i.p.), C—control group, BK—bradykinin postconditioning 48 h after ischemia, 3D—ischemia followed 3 days of reperfusion, 7D—ischemia followed 7 days of reperfusion, 28D—ischemia followed 28 days of reperfusion. Time necessary to enter the box is expressed in seconds

**Fig. 4:**
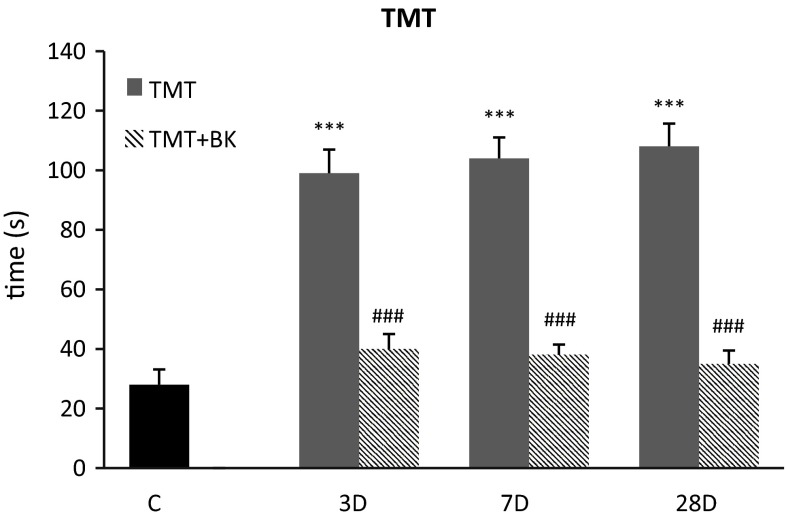
Behavioral results using Barnes Maze for experimental groups after application of trimethyltin and with use of delayed postconditioning. The latency to enter the box under the surface of the platform was measured in all experimental groups. Mean ± SD, *p* < 0.05 (*, #), *p* < 0.01 (**, ##), *p* < 0.001 (***, ###); *value compared to control, #value compared to ischemia without bradykinin postconditioning, C—control group, BK—bradykinin postconditioning 48 h after ischemia, 3D—TMT injection followed 3 days of reperfusion, 7D—TMT injection followed 7 days of reperfusion, 28D—TMT injection followed 28 days of reperfusion. Time necessary to enter the box is expressed in seconds

After the behavioral training and following testing the average time to reach the escape box after 10 min of global ischemia and 3 days of reperfusion was 130 s. After 7 days of reperfusion the measured time was 135 s and after 28 days of reperfusion it was 142 s, which was significantly increased in comparison with the control (28 s). The application of bradykinin postconditioning reduced the average latency in every group, causing it to nearly reach the control value. In the first group with 1 day of reperfusion after postconditioning we measured a latency of 29 s; in the second group after 5 days of reperfusion 39 s and in the third one after 26 days of reperfusion it was 36 s (Fig. 3). After chemical insult caused by TMT injection and 3 days of reperfusion was an average time of 99 s was measured. In the group with 7 days of reperfusion the time was 104 s and with 28 days of reperfusion it was 108 s. When bradykinin was used as a postconditioner in the first group, it reduced the average time to 40 s, in the second group to 38 s and in the third one to 35 s (Fig. 4).

### Fluoro Jade B Staining and NeuN Immunostaining

**Fig. 5:**
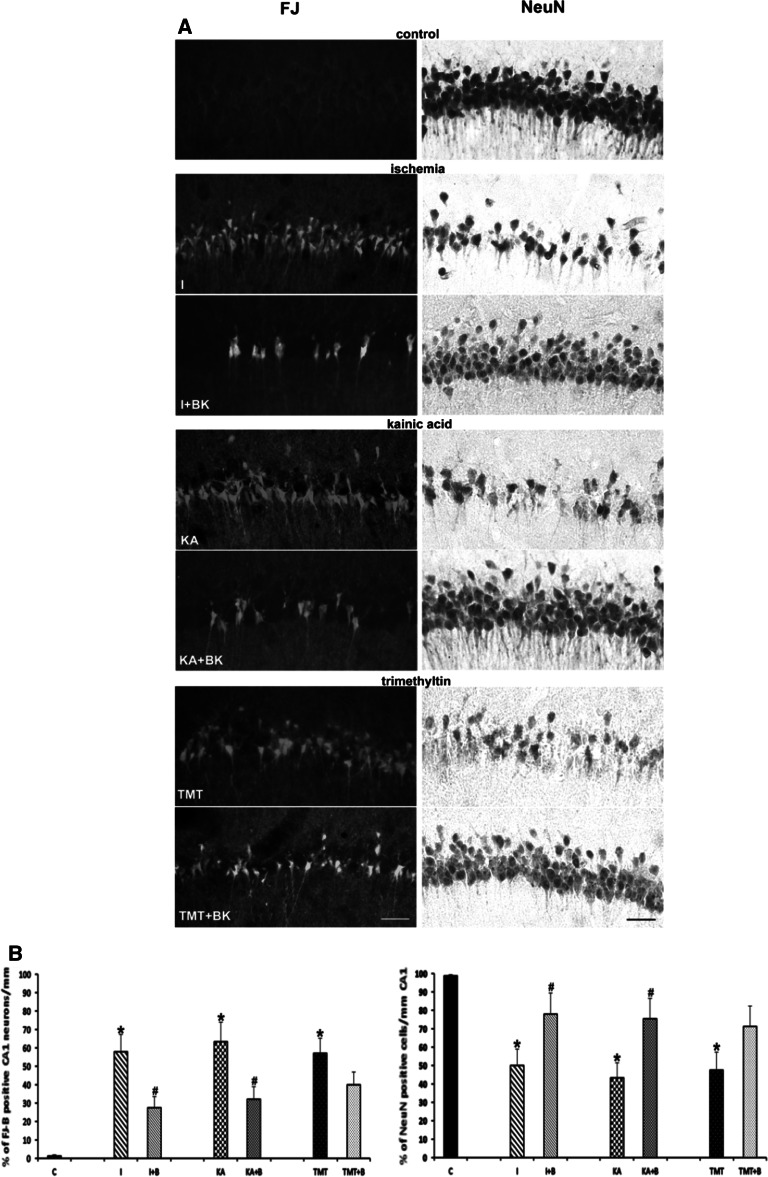
Photomicrographs of the CA1 area of hippocampus. Neurodegeneration is visualized by Fluoro Jade B staining (FJ), surviving neurons are visualized by NeuN immunostaining. Visualized neurons are of control, 10-min ischemia and with bradykinin (BK) postconditioning (150 µg/kg i.p.), Kainate injection (KA, 8 mg/kg i.p.) and with BK postconditioning, TMT injection (8 µg/kg i.p.) and with BK postconditioning. **a** Magnification is expressed by *scale bar* = 50 µm and applies to all photomicrographs. Quantification of fluorescence intensity (% of control) in the *left column* and the number of NeuN+ cells in the *right column* (**b**). Values were taken as a mean ± SD of six animals in each group. *p < 0.05 compared to control, #*p* < 0.05 compared to ischemia without application of bradykinin

Fluoro Jade B staining was used for visualization of neurodegeneration. It showed a significant difference between the damage of CA1 pyramidal neurons induced by 10 min of ischemia and 7 days of reperfusion and following the use of bradykinin as postconditioning 2 days after the same interval of ischemia (Fig. 5 left column). NeuN antibody labels nuclei and cytoplasm of most neuronal cell types in all regions of the adult brain. In control hippocampal sections NeuN immunoreactivity was present in the pyramidal cells of CA1 and also in granular cells of dentate gyrus. In the experimental group subjected to 10 min of ischemia and 7 days of reperfusion without any postconditioning significant changes in NeuN immunoreactivity were found in the most vulnerable pyramidal cells of CA1 region (Fig. 5 right column). Quantification of fluorescence intensity shows its increase in the ischemia group (57.81 %) and decrease after BK postconditioning (27.53 %). The effect of postconditioning is visible also in the groups with chemical intoxication. After KA injection the percentage of FJ positive neurons was 63.51 % and following BK postconditioning it was only 32.21 %, and in TMT groups it was 56.98 % and after BK postconditioning only 40.01 %. The percentage of NeuN positive neurons was 50 % after 10-min of ischemia and 78.04 % in the group with BK applied. After KA intoxication we measured 43.24 % of surviving cells and after BK postconditioning 75.67 %, after TMT it was 47.63 % and after BK postconditioning the amount increased to 71.28 % (Fig. 5b).

## Discussion

Since brain relies exclusively on blood-borne glucose for its energy source, any interruption in cerebral blood flow will lead rapidly to energetic failure and a dramatic fall in intracellular levels of ATP. The consequences of this will be both an increase in the concentrations of extracellular glutamic acid and a sensitization of neurons. Also, oxygen deprivation plays an important role in brain damage. Inadequate blood flow to the brain leads to reduced oxygen and prolonged hypoxia induces apoptotic cell death. It is known that during ischemia and following reperfusion damage, an increased amount of glutamate is released into the extracellular compartment of the cells and participates in the pathogenesis of ischemic lesion. The accumulation of excitatory amino acids (EAA) in extracellular spaces is due to their increased release but also to a decrease in their reuptake [42]. At the core of ischemic injury, glutamate is released at very high concentrations, approximately 80 times the baseline level [43]. Glutamatergic overstimulation of postsynaptic NMDA receptors leads to subsequent calcium overload, which precipitates the delayed cell death of hippocampal CA1 neurons after global cerebral ischemia (GCI) [44]. During the acute phase of cerebral infarction, the levels of glutamate are elevated. Under normal circumstances, glutamate does not cross the blood–brain barrier [45]. The correlation between EAA levels in CSF and in plasma suggests diffusion through the blood–brain barrier. The neurotoxic effects are of greater importance [46]. The increase of blood glutamate after ischemia has been studied in both focal and global ischemia models. In the focal ischemia model, the glutamate concentration was elevated during the entire reperfusion period and also in the forebrain ischemia model it remained elevated during the 48 h period after insult. In both cases, the induction of ischemic tolerance by postconditioning and application of a second stress reduced the concentration of blood glutamate to the control levels [12]. It has been observed, that mild activation of NMDA-receptors by subtoxic doses of NMDA agonists confers ischemic tolerance to neurons. Furthermore, there is growing evidence that under certain conditions, activation of synaptic NR2A receptors, may exert a prosurvival role in neurons, while activation of extrasynaptic NR2B receptors may promote cell death [47, 48]. Also, the enzyme glutamine synthetase (GS) is expressed in glial cells and may affect glutamate excitotoxicity. Up-regulation of GS expression after ischemia may constitute a neuroprotective mechanism [11].

During the processes of ischemic cascade and with the involvement of ischemic pre and postconditioning, the opening of the mitochondrial ATP-dependent potassium channel (mitoKATP) has been described [49, 50]. Involvement of the mitoKATP pathway in cerebral anesthetic-induced postconditioning has been found [51]. Ischemic postconditioning may also be mediated by the mitoKATP channel as in cerebral ischemic preconditioning [52]. Mitochondria appear to be central in the postconditioning process, possibly because the opening of mitoKATP induces production of ROS or prevents calcium overload. A beneficial effect of cerebral postconditioning is the recovery of mitochondrial membrane potential independently of any effect on oxidative phosphorylation. Another relevant consequence of mitoKATP opening is the inhibition of apoptosis [53, 54]. Interestingly, the number and duration of occlusion–reperfusion cycles determined the level of neuroprotection suggesting that the effect of postconditioning depend on both the number and duration of occlusion and reperfusion cycles.

Effect of BK as a stressor usable for a preconditioning is known from early 90-th from studies in myocardial ischemia [55–57]. Equally as a majority of other stressors it can be used in both pre- as well as post-conditioning and in any ischemic tissues.

Cerebral protection through ischemic preconditioning is widely achieved in many models of global and focal cerebral ischemia. Ischemic preconditioning is the induction of tolerance to noxious ischemia through pretreatment with exposures to mild ischemia. Among other effects of preconditioning, a reduction in glutamate excitotoxicity during ischemia contributes to anti-ischemic effects, as preconditioning decreases glutamate release and promotes glial glutamate uptake [58, 59]. Ischemic preconditioning activates those responses seen in anoxia/ischemia tolerant organisms whereas preconditioning with a noxious agent such as endotoxins may protect against ischemia by modulating inflammatory pathways [60]. The activation of bradykinin B2 receptor induces the release of inflammatory mediators such as reactive oxygen radicals and other agents [61, 62] which leads to vasodilation, increase of vascular permeability, blood–brain barrier disruption, and cerebral edema [63]. B2 receptor blockade also showed positive results in a focal cerebral ischemia model. It improved neurological outcome, reduced infarct volume, and attenuated the development of brain edema [20, 64]. Postischemic blockade of bradykinin B2 receptors at the time of reperfusion improved neurological outcome and inhibited infarct evolution without influencing cerebral blood flow in a rat model of temporary focal cerebral ischemia [65]. It is known that B2 receptor in astrocytes is involved in Ca2+ signal transmission from astrocytes to neurons during stimulation with BK in mouse astrocyte–neuron co-cultures. This is because of the activity of VSOR (volume-sensitive outwardly rectifying anion channels) in the pathway for bradykinin-induced glutamate release. BK stimulates B2 receptors, independent Ca2+ rise and also the Ca2+-independent generation of ROS in astrocytes. ROS then activates glutamate-VSORs without inducing swelling of astrocytes, thereby causing the release of glutamate. This activates NMDA receptors, leading to an increase in the cytosolic Ca2+ concentration of neurons. The signals transmitted from astrocytes by this pathway are likely to be involved in the anti-apoptotic roles of bradykinin in neurons [66].

We observed in our experiments that selectively vulnerable hippocampal CA1 regions are able to recover from the ischemic insult and obtain the ischemic tolerance. In this process, the presence of a second pathophysiological stress applied before the beginning of the irreversible stage of delayed neuronal death is critical. In the model of 10-min global ischemia, we measured considerably increased levels of glutamate. This was present after toxic TMT damage as well. We also examined the intensity of neurological defects and the damage of hippocampal spatial memory. Bradykinin, used as a second stressor, proved to be a useful tool for initialization of endogenous defense mechanism. Delayed postconditioning can be used effectively after some apoptosis-inducing intoxications [67]. The levels of glutamate in brain tissue changed greatly after the sublethal stress that followed the lethal one. The reduction of the ischemic damage was confirmed by our Fluoro Jade B staining and Neu N immunoreaction. In some experimental groups, the reduction was so great that the values were lower than the control. The ischemic tolerance was a useful tool for preventing ischemic damage not only in groups with a short-term survival pattern but also in groups with a longer pattern. Delayed postconditioning prevented the irreversible damage of neurons in CA1 area of hippocampus. It is interesting that glutamate after ischemia without conditioning increases equally in both CA1 as well as DG but neurons in DG survive such situation while CA1 sucumb to delayed neuronal death. Also, the behavior of animals and their spatial orientation improved significantly. The animals after bradykinin postconditioning performed the test well relative to the groups without postconditioning. It was already known that neuroprotection induced by immediate and delayed postconditioning provided overall improvement in neurological function up to 1–3 months after prolonged ischemia [68, 69]. This was shown by using a wide array of behavioral tests for evaluating motor function or spatial learning and memory. With the water maze assessment, better memory and spatial learning performance 3 weeks after global ischemia in rats subjected to immediate postconditioning was observed [70]. The Post C neuroprotection was correlated with an improved spatial learning/memory outcome after GCI [13].

## Conclusions

Long-term studies have shown that postconditioning reduces ischemic damage and infarction after lethal ischemia, and it has also been demonstrated that it improves animals’ neurobehavioral functions and memory performance. Our study demonstrates that delayed postconditioning with bradykinin application 2 days after the lethal insult can induce ischemic tolerance and protect the selective vulnerable neurons against the irreversible damage. The behavioral test used in our experiments confirms also the memory improvement in experimental animals. Postconditioning seems to reduce ischemic injury possibly by blocking the overproduction of ROS and lipid peroxidation. The use of bradykinin as a postconditioner before the irreversible changes of I/R occurred can inhibit apoptosis. Also, important to mention is involvement of other processes that induce the activation of prosurvival actions of neurons such as mild activation of NMDA-receptors or the actions of mitoKATP pathway in the processes of postconditioning. However, future studies are necessary to fully understand the protective mechanisms and improve the therapeutic strategies.
